# The application of a biometric identification technique for linking community and hospital data in rural Ghana

**DOI:** 10.3402/gha.v9.29854

**Published:** 2016-03-17

**Authors:** Eliezer Ofori Odei-Lartey, Dennis Boateng, Samuel Danso, Anthony Kwarteng, Livesy Abokyi, Seeba Amenga-Etego, Stephaney Gyaase, Kwaku Poku Asante, Seth Owusu-Agyei

**Affiliations:** Kintampo Health Research Centre, Kintampo, Ghana

**Keywords:** biometrics, fingerprint, identification, techniques, electronic, database, data-linkage

## Abstract

**Background:**

The reliability of counts for estimating population dynamics and disease burdens in communities depends on the availability of a common unique identifier for matching general population data with health facility data. Biometric data has been explored as a feasible common identifier between the health data and sociocultural data of resident members in rural communities within the Kintampo Health and Demographic Surveillance System located in the central part of Ghana.

**Objective:**

Our goal was to assess the feasibility of using fingerprint identification to link community data and hospital data in a rural African setting.

**Design:**

A combination of biometrics and other personal identification techniques were used to identify individual's resident within a surveillance population seeking care in two district hospitals. Visits from resident individuals were successfully recorded and categorized by the success of the techniques applied during identification. The successes of visits that involved identification by fingerprint were further examined by age.

**Results:**

A total of 27,662 hospital visits were linked to resident individuals. Over 85% of those visits were successfully identified using at least one identification method. Over 65% were successfully identified and linked using their fingerprints. Supervisory support from the hospital administration was critical in integrating this identification system into its routine activities. No concerns were expressed by community members about the fingerprint registration and identification processes.

**Conclusions:**

Fingerprint identification should be combined with other methods to be feasible in identifying community members in African rural settings. This can be enhanced in communities with some basic Demographic Surveillance System or census information.

## Introduction

In public health, there is increasing interest in the availability of reliable data from general community that can be linked with health facility information (1). Integrating community and health facility data promises reliable counts that can be generated as numerators and denominators of the population and used in generating outputs for health planning and policy formulation such as disease burdens, spread of diseases, and health service coverage (2). The success of this data linkage is determined by the accuracy of identifying individuals and matching their basic demographic information with their data at health facilities where they seek care (3). A method for correctly identifying individuals must also ensure that the privacy of that individual's data is preserved (4).

In the developing world, the main challenge in matching population-level and health facility data is the lack of reliable and shared identification methods (5, 6). The combination of biographic data and national identification numbers from health insurance cards, passports, and birth certificates is predominantly used to identify patients (7).

Health institutions in Ghana combine the use of national health insurance scheme (NHIS) numbers with biographic data and locally generated hospital IDs to identify those who seek care at the health facility (8). However, one common challenge is that patients who attend health facilities fail to present consistent identification, either because they have misplaced what they possess or forgot to carry their identification cards with them to seek care (5). Furthermore, most hospitals in poor resource settings operate with paper-based systems, making it commonplace to find one identification associated with multiple people (9). These challenges result in patient-diagnosis mismatch, duplication of patient records, and incomplete reporting, which potentially compromises the reliability of data for decision-making (10, 11). Moreover, multiple forms of identification using personally identifiable information tend to compromise data security in information systems, which can increase the risk of identity theft (12).

This manuscript reports experiences of using biometric identification techniques in a routine health information process at the Kintampo North Municipal Hospital and Kintampo South District Hospital (KSDH) in the central part of Ghana. The experiences reported here are ancillary to the data linkage project initiated as part of the INDEPTH Network Effectiveness and Safety Studies (INESS), which provided a platform for researchers in Africa to conduct Phase IV trials on a large scale (13, 14).

## Biometric identification

Biometric identification is a method of recognizing an individual, using a physical or behavioral characteristic (15). Common among such include systems for fingerprint identification, face recognition, iris scan, retina scan, hand geometry, and voice scan (16, 17). The use of biometric technology has been successful in the fields of access control, attendance monitoring, and securing health data, resulting in the rapid advancement of this and related technologies (18–25). Compared to other biometric identification systems, fingerprint identification has a very large vendor base, with different templates and algorithms (26, 27). This potentially makes fingerprint technology relatively affordable for resource-poor settings.

Fingerprint identification is based on the notion that patterns on the frictional fingertips of an individual are infallible proof of identity (28–30) and that these patterns remain unchanged throughout life. Patterns on the sole of the foot can also serve as identity (31). A sensor is used to capture or detect a fingerprint. Software programs commonly use either minutiae-based matching or pattern matching for identification (32, 33).

## Methods

### Study setting

The study was conducted in the Kintampo North and South Districts, which are located within the forest savannah transitional ecological zone (34). The Kintampo Health Research Centre operates a health and demographic surveillance system, which routinely collects longitudinal demographic data such as pregnancies, births, deaths, migrations, and determinants of health (including household characteristics, socioeconomic conditions, and education) of a well-defined population with each individual identified by a unique number (35). The Kintampo Health and Demographic Surveillance System (KHDSS) has a population of 140,000. There are 12 health centers and 30 community-based health planning services that provide health services to the people. Two district hospitals serve as patient referral points within the study area and approximately 50% (66,508) of the resident population are within the catchment area of the two hospitals.

### Study procedure

A mass field enrollment exercise was undertaken from January 2010 to June 2011. This exercise was to expand data the electronic database (EDB) of the KHDSS to include the biometric (fingerprint) data and photographs of all resident members. Resident members in the KHDSS communities were notified and comprehensively educated about the enrollment exercise. Approaches to education and awareness included radio announcements, the use of announcement vans to communicate in local dialects, and the use of religious and information centers. During enrollment, all 10 fingerprints and a photo of each resident member were entered into the database. For infants 2 years and below, heel prints were acquired instead and their photos were taken as a mother–child pair. All enrolled members received identification cards.

#### Recruitment of field team

Twenty community members with post-high school qualification and basic computer skills were trained to acquire the fingerprints and photos of the residence members and to enter them into a specially designed database application. The communities were divided into 10 zones. Ten field teams were formed, with two members in each team. Each team was given a complete set of the tools needed for the exercise, which included field notebooks and a problem sheet to record exceptional incidences and problems that occurred during the enrollment exercise. Raincoats were also provided to protect the delicate equipment from unexpected rains during the enrollment exercises. After the mass enrollment exercise, two teams were maintained to conduct periodic updates on individuals who newly joined the surveillance system. A data manager was assigned the task of updating the EDB of the KHDSS with the data collected from the field on daily basis.

#### Health facility system setup

Following the mass enrollment, a desktop computer was set up at each of the out-patient department (OPD) units of the two district hospitals, Kintampo North Municipal Hospital and the KSDH. Each computer was equipped with a fingerprint device and a handheld barcode reader. The upgraded KHDSS EDB used to enroll resident members was installed on the computers at the OPD with the purpose of identifying and retrieving the records (FOLDER) of resident members who visited the hospitals. Hospital staff at the OPD were trained to use the fingerprints and other techniques to identify members that attended the hospital for care.

#### Identification process at the hospital

**Fig. 1:**
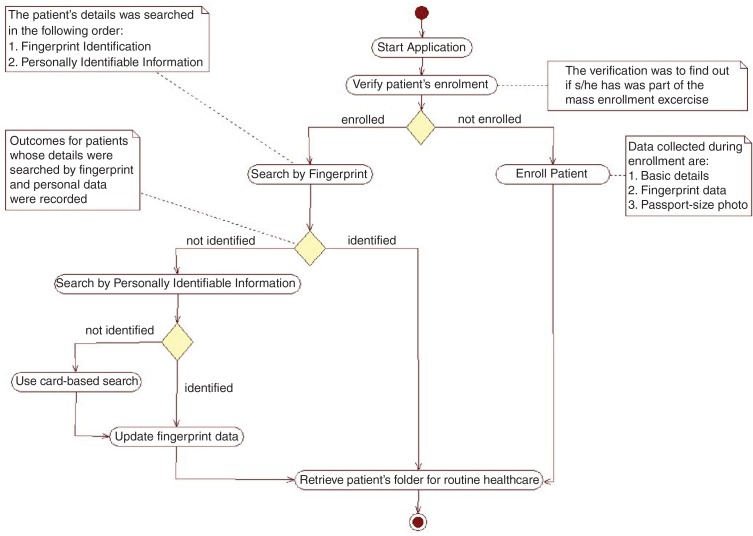
Activity diagram for the identification process. The solid circle at the top of the flow diagram indicates the start of the process. The colored rectangles with curved edges indicate the major steps in the activity. The diamond shapes in the flow diagram represent points of decision in the activity; the arrows show the sequence and directions of the activity. The plain (white) rectangles provide more explanation for some of the processes. These plain rectangles are linked to the processes they explain by dotted lines; the solid circle with a white ring around it indicates the end of the activity.

Only individuals who visited the OPD during routine working days (Monday to Friday) between 08:00 and 17:00 Greenwich Mean Time were processed with the EDB. During this period, the EDB was used to record the date of visit for each individual that was identified. The EDB was also designed to record data from non-resident members who attended the hospitals. The residence status of individuals was confirmed by residence information on the EDB. The order of identification for the methods employed is shown in Fig. 1. The first is by fingerprint, which is then followed by using a combination of personally identifiable details such as name, address, age, and community of residence. Those who were not identified by fingerprint but were identified by other methods were recorded as *false negative* for fingerprint identification. True and false positives were confirmed by membership details retrieved, which included the field-based photographs taken during the enrollment exercise. A confirmed match of the details was considered a *true positive* and if the wrong details were retrieved it was considered a *false positive*.

To minimize the impact of disruption in the existing hospital process, which may result in delays and long queues, the hospital staff advised that resident patients who were not part of the enrollment exercise be excluded from the current study. However, a separate process was set up to enroll these patients and their basic details including NHIS ID (if available), fingerprints and photos were subsequently updated as shown in Fig. 1.

#### Mobilization of equipment and software setup

**Fig. 2:**
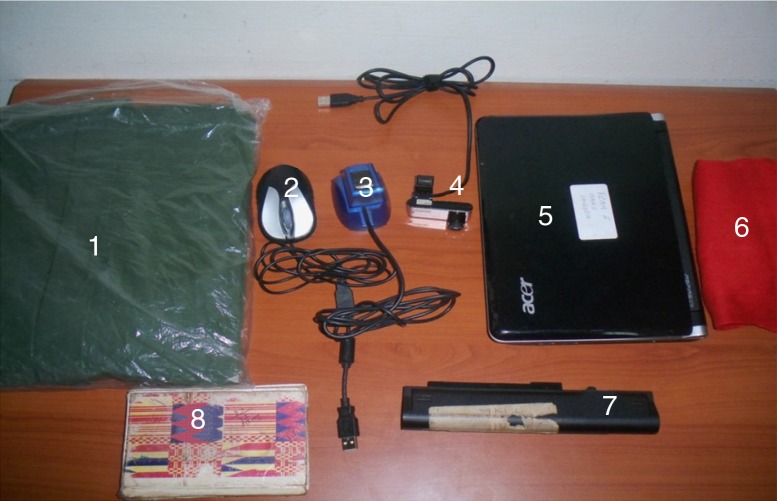
Computer hardware and logistics: (1) the raincoats; (2) the mouse; (3) the biometric fingerprint devices; (4) the web cameras; (5) the mini-laptops; (6) the red calico used for the background of the photos taken; (7) the spare batteries for the mini-laptops; and (8) the field notebooks used.

**Fig. 3:**
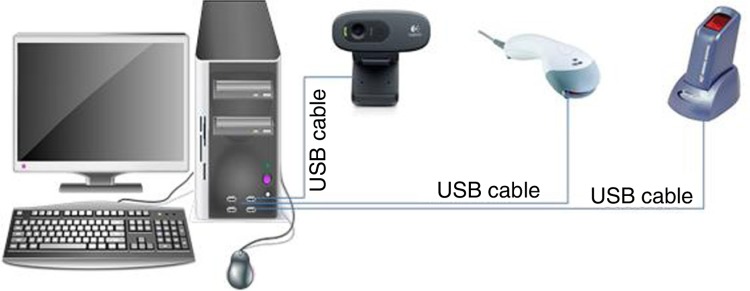
Setup for the computers, with peripheral devices. This setup is typical of computers at the health facilities. Each of the peripheral devices was connected to the computer system's unit via a USB cable. The device to the extreme right of the diagram is the fingerprint detector. Next to the fingerprint detector is a barcode reader for scanning identification cards with barcodes. The last device (black) is a web camera.

Ten mini-laptops were used for the enrollment exercise; each laptop was equipped with peripheral devices (Figs. 2 and 3) to facilitate fingerprint registration for the entire residence population. In addition, a license key was purchased to activate the fingerprint scanning device of each computer and a server was purchased to store backups. One box of tools and other accessories were procured for the maintenance and repair of the computers.

As shown in Fig. 2, Acer Aspire One 2007 (California, USA) mini-laptops were used. They had a memory size of 1 gigabyte (GB) and a hard disk capacity of 160 GB. Logitech HD Webcam C310 2010 (California, USA) devices were used, as well as the 2008 version of the Hamster plus IV fingerprint device, developed by SecuGen Corporation (California, USA). In addition, a USB (universal serial bus) mouse was attached to each laptop to improve speed in navigation.

The biometric software for the health facilities was developed with the Java Development Kit version 1.5 (Oracle Corporation, 2010; California, USA). The MySQL Database Community Edition 5 (Oracle Corporation, 2010) was used to design the database for the application. In addition, the Griaule Fingerprint SDK 2009 (California, USA) library was used to connect the software to the fingerprint device. The fingerprint library had a threshold of 45 and a rotation tolerance of 180 (36). Figure 3 shows how the software and hardware systems were set up for patient identification at the health facility.

## Results and discussions

The average time it took a team to confirm residence status and enroll an individual was 30 min. An average of 26 enrollments were done per team per day. Factors such as sudden computer hardware or software malfunctions and requests for further clarifications prolonged the time spent on enrolling some individuals. The mass enrollment covered 83.4% (117,403/140,000) of the resident members. However 100% coverage was achieved at the catchment area of the two district hospitals. The catchment areas are defined by district health directorates. These definitions are based on several factors including population threshold and administrative and political reasons. None of the resident members refused to enroll their fingerprints and photographs. This may be due to the comprehensive education program that was developed and implemented by a dedicated team prior to and during the enrollment.

At both hospitals, 43,917 visits were registered in the EDB during the 12-month period. Of this number, 27,662 visits (63%) were linked to resident members. It is worth noting that by virtue of their location along a major highway linking the north and south of Ghana, a large number of non-resident members attend these district hospitals for care. A total of 10,214 out of the 27,662 visits were members identified by ID card (either hospital or NHIS), who were therefore excluded from our analysis. Our analysis is thus based on 17,448 of the 27,662 visits. During the identification process at the hospitals, no refusals were recorded in the use of fingerprints to identify the patients that attended the health facilities. It took approximately 7 min for the staff to record biographic data, fingerprints, and a passport-size photograph from a patient whose data had not been recorded during the mass enrollment exercise. However, fingerprint identification of an enrolled patient took 30 sec on average. It is also worth noting that in addition to the 16,255 non-residents enrolled, 438 of the visits were by members who were non-resident prior to the mass enrollment exercise and therefore not enrolled.

**Fig. 4:**
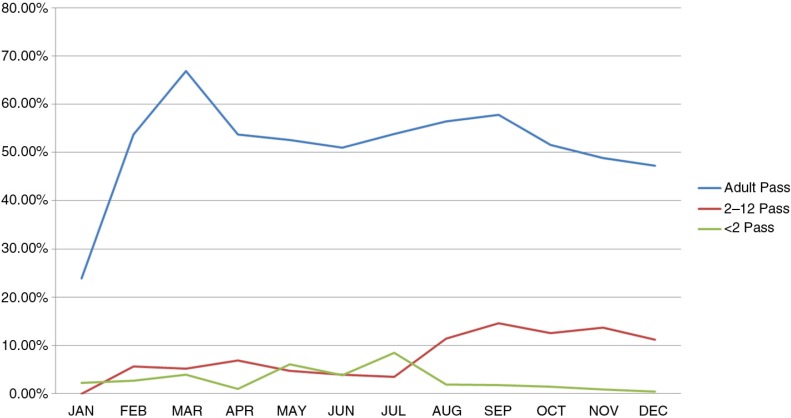
Age grouping for visits successfully identified by fingerprint for the year 2012. The x-axis categorizes the number of successful identifications by month. The values on the y-axis are percentage rates over the total number of visits searched by fingerprints for the given month. The bar graphs indicate the percentage of patient visits from three age groupings for which fingerprint identification was successful. The blue bar graphs represent age groupings from 13 years and above, the red bars represent age groupings from 2 years to 12 years, and the green bars represent age groupings of children under 2 years.

As shown in Table 1, out of the 17,448 visits from resident members, the fingerprint method successfully identified and linked 11,465 but was unable to identify 5,983. This translates to a sensitivity of 65.7 and 100% specificity, as demonstrated in Table 2. Fingerprint identification was further analyzed among three age groups of resident members (Fig. 4). Results from both health facilities indicated that the fingerprint identification technique achieved an average of 68.7% success in accurately identifying individuals aged 13 and above. An average of 57.3% was achieved among children from ages 2 to 12 years while a 35.2% average success rate was achieved among infants less than 2 years old. The residents of the area are mainly engaged in farming and manual work (34), which may have caused poor quality fingerprints (37, 38). This factor may explain the relatively low success rate for fingerprint identification among adults. Very low rates were also realized in identifying the fingerprints of the very young in the population; those between ages 0 and 12 years posed the main challenge. This confirms what is known in the literature, suggestive of the fact that the fingerprints of children in this age group are still developing, thus making it inconsistent for use in identification (39).

**Table 1:** The confusion matrix of the fingerprint, and other identification tests

	Enrollment condition	
	
Identification test results	Enrolled	Not enrolled
Fingerprint		
Identified	11,465	0
Not identified	5,983	438
Other personal information		
Identified	3,440	93
Not identified	2,450	438
Fingerprint+other personal information		
Identified	14,905	93
Not identified	8,433	438

**Table 2:** Sensitivity and specificity of fingerprint and other identification test

Test	Sensitivity (%)	Specificity (%)	Precision (%)
Fingerprint identification	65.7	100.0	100.0
Other personal identification	57.6	82.5	97.4
Fingerprint+other identification	85.3	82.5	99.4

Further linkage was done based on the personal identifiers described above on the 5,983 for which fingerprint identification failed. As shown in Table 1, 3,440 of these visits were ultimately successfully identified, whereas 2,450 of the visits were unsuccessfully identified. Additionally, 93 visits resulted in identification of the wrong person. These data result in a sensitivity of 57.6% and specificity of 82.7% as shown in Table 2. There were issues surrounding the use of the personal information–based search method, which resulted in the low success rate. Due to the relatively low educational levels among residents (34), members provided inconsistent name spellings and were less able to remember details like their age and date of birth as compared to their communities of residence. In other situations, members had migrated away from the communities where they were enrolled. These issues affected the precision of the information-based search.

The combined effect of using both fingerprints and personal information to identify patients resulted in successfully linking 14,905 of the total visits under consideration. This effort achieved a sensitivity of 85.3% and a specificity of 82.5% (Table 2). This finding suggests that biometric identification can be a useful supportive mechanism to mitigate the limitations associated with other methods of identification. Our findings further suggest that adding photo verification to other personal details is a very advantageous technique in confirming the true identity of individuals.

## Conclusions

This study has demonstrated that it is feasible to use biometrics to support other methods of patient identification in linking health facility data to population data in predominantly rural communities where a health and demographic surveillance system exists. However, such an undertaking is accompanied by several challenges, including comprehensive education strategies, high capital investments in technology and maintenance, and very poor fingerprint sensitivity due to occupational and other socio-demographic characteristics. Though the biometric method has relatively low sensitivity, it will be very useful as an ancillary to card-based and information-based identification techniques. Moreover, the applicability of this system in places without a demographic surveillance system needs to be investigated. These factors may require context-specific approaches when deploying this technique in other settings.

Due to resource constraints, the study was conducted at 2 out of 36 health facilities within the Kintampo surveillance site. Similar studies should consider a wider coverage of health facilities to provide more representative results of an entire residence population.

Further work is also needed to assess the extent to which fingerprint identification reduces challenges associated with patient-diagnosis mismatching, as well as issues of duplicated and fragmented patient data. Further studies should also examine other essential determinants of adopting biometrics in low-resource settings, which may include the ease of using the fingerprint devices in routine healthcare processes, how effective biometric identification is during emergency cases, as well the capability and willingness of available information technology (IT) support staff to maintain such systems.

On a production scale, data linkage with biometrics in resource-limited settings may consider the adoption of low-power technologies with energy harvesting capabilities to minimize the effects of frequent power outages on its implementation (40).
